# Peri-Implant Bone Loss at Implants Placed in Preserved Alveolar Bone *Versus* Implants Placed in Native Bone: A Retrospective Radiographic Study

**DOI:** 10.2174/1874210601812010529

**Published:** 2018-07-31

**Authors:** Johann Bui Quoc, Aurélie Vang, Laurence Evrard

**Affiliations:** Department of Dentistry, Oral and Maxillofacial Surgery, Orthodontics, Stomatology, Erasme Hospital, Université libre de Bruxelles, Brussels, Belgium

**Keywords:** Allograft, Alveolar preservation, DFDBA, Platelet concentrates, PRF, Mandibular canal

## Abstract

**Objectives:**

The aim of our study was to compare peri-implant bone loss at implants placed in alveolar sockets filled with a particulate allogenous bone graft (DFDBA 300-500 µm) and platelet concentrates *versus* at implants placed in the native bone.

**Materials and Methods:**

A retrospective clinical study was performed. A total of 84 patients were included with 247 implants for the restoration of mono and pluri-radicular teeth: 169 implants in native bone and 78 in socket-grafted bone. The peri-implant bone loss was measured by 2 independent operators at 6 and 12 months.

**Results:**

The overall mesial and distal peri-implant bone losses were 0.9 ± 0.7 mm and 0.9 ± 0.8 mm at 6 months, respectively, and 1 ± 0.65 mm and 1.2 ± 0.9 mm at 12 months, respectively. In the tested group, the bone loss was 0.8 ± 0.8 mm at 6 months and 1.2 ± 0.9 mm at 12 months. In the control group, the bone loss was 1.0 ± 0.7 mm at 6 months and 0.95 ± 0.6 mm at 12 months. There were no statistically significant differences in bone loss between the two groups. Taking both groups together, there were no statistically significant difference in bone loss between patients with or without histories of periodontitis, but there was a statistically significant difference in bone loss between the mandible and maxilla as well as between unitary and total edentations and between partially and total edentulous patients.

**Conclusion:**

At 6 and 12 months, the peri-implant bone loss in sockets preserved with DFDBA and platelet concentrates was similar to the peri-implant bone loss in native bone.

## 1 INTRODUCTION

Currently, dental implants are regularly included in the overall treatment plan for patients. Implant success is defined by criteria that have changed over time. The criteria commonly accepted in implantology were originally defined by Albrektsson and colleagues in 1986 [1].

According to these authors, the individual implant had to be immobile when it was clinically tested, the radiography could not show a radiolucent space around the implant, the bone loss had to be less than 1.5 mm after the first year of setting function and 0.2 mm per year thereafter, the implant had not to be responsible for persistent and/or irreversible signs and symptoms, such as pain, infection, nerve damage, paresthesia or penetration into the mandibular canal, and it was necessary that the minimum success rate of implants was 85% at the end of a period of 5 years and 80% at the end of a period of 10 years [1].

These success criteria were majored by Smith and Zarb [2]: the implant should not cause damage to the roots of adjacent teeth or perforate the mandibular canal or maxillary sinus or nasal cavity. The authors indicated also that the absence of radiolucent edging on the mesial and distal sides of the implant did not exclude the possibility of bone loss or lack of osteointegration on vestibular and/or lingual parts. They stated also that bone loss should be less than 1.5 to 1.6 mm at the end of the first year of function and 0.2 mm per year thereafter.

In a more recent article [3], the authors emphasized that, to indicate success, periodontal criteria should be carefully considered: peri-implant pocket depth should be less than 3 mm at probing, and there should be an absence of blood and/or pus on probing, an absence of swelling and receding gums, a low plaque index and a width of the attached mucosa greater than 1.5 mm. For other authors [4, 5], peri-implant pocket depth should be less than 5 or 6 mm to indicate success. Although gingival inflammation (mucositis) is not an implant failure criterion, it is nevertheless important to treat it so that it does not develop in peri-implantitis causing peri-implant pockets and bone loss [2, 4]. Compared with initial papers that were focused on success, the aesthetic and personal evaluation of the patient’s appearance with his or her prosthesis has become one of the most important success criterions [3]. It has been said that, in the future, a common index of aesthetic criteria should be established by clinicians [2, 3, 6].

Our study aimed to evaluate peri-implant bone loss over time, which is known to be influenced by various factors. Hygiene is the preponderant factor. The accumulation of plaque around implants is responsible for inflammation of peri-implant soft tissue, which can lead to bleeding and/or pus on probing and bone loss.

Therefore, the practitioner must ensure adequate forms of supra-structures to allow for easy cleaning to ensure long-term implant preservation [7-12]. Patients with a history of periodontal disease or with active periodontitis have increased the risk of peri-implantitis and therefore of bone loss. It is therefore essential to treat periodontitis prior to implant placement to avoid bacterial translocation [7, 9-12].

In several studies, there has been a correlation between the consumption of tobacco and marginal bone loss. Thus, it is important to encourage patients to stop smoking before they receive their implants [7, 9-12]. Studies aiming to determine if there is an association between diabetic patients with poor glycemic control and the occurrence of peri-implantitis have reported contradictory results [7, 9, 12]. Nevertheless, there is a consensus on the fact that subjects with a good metabolic control remain at low risk for implant failure [7, 9, 12]. Also contradictory have been the results of studies on the relationships between the roughness of implants and bone loss [7, 12]. There is a link between daily consumption of more than 10 g of alcohol per day and loss of peri-implant marginal bone [6, 7]. Other factors that could have an influence on peri-implant bone are endodontic infections on neighboring teeth [6, 13] and occlusal overload [10-12].

Anaerobic gram-negative bacteria grow around the implant and their interaction with the biofilm can lead to their aggregation and to the destruction of the peri-implants tissues. When a peri-implantitis is diagnosed, a higher amount of micro-organisms is present and most of them are anaerobic [12, 14, 15].

Tooth extraction is followed by physiological alveolar bone resorption, which is irreversible and can reach up to 40% in height and 60% in width with great loss occurring within the 3 months after extraction [16]. Insufficient bone can compromise dental implant treatment with a risk of injuring anatomical structures [17]. Therefore, adequate alveolar ridge preservation is essential for aesthetical outcomes and correct implant placement [18].

Among the biomaterials used for post-extraction alveolar filling [19-23], allogenic bone has been described as a suitable material. In the particulate form, Freeze-Dried Bone Allograft (FDBA) and Demineralized Freeze-Dried Bone Allograft (DFDBA) have been used in dental surgery and alveolar ridge preservation technique [23-27]. It has been shown that, when used in post-extraction sockets, allografts have positive effects on height preservation [23, 24]. In a histological study of alveolar preservation [26], it was shown that DFBDA led to a statistically significantly greater mean percentage of newly formed vital bone than FDBA.

Platelet concentrates (platelet-rich-fibrin) are obtained by centrifugation of blood, following a method first described by Choukroun and colleagues [28]. These materials contain high concentrations of growth factors [29] (PDGF, TGF-β, IGF and VEGF), and inflammatory molecules (IL-1β, IL-4, IL-6 and TNF-α), and they could enhance the healing process [30], possibly leading to better bone repair and regeneration [30, 31]. It has been shown that platelet concentrates accelerate the healing of dermal soft tissue [32] and of the oral mucosa in cases of extraction [33, 34]. It remains unclear whether they are able to accelerate bone healing and influence the bone quality of extraction sockets, although it has been suggested in some studies [35, 36]. In oral surgery, the benefits in the treatment of periodontal defects with a combination of platelet concentrates and DFDBA have been shown [37].

To our knowledge, there is no previous study comparing peri-implant bone loss at implants placed in preserved sockets with DFDBA and platelet concentrates and peri-implant bone loss at implants placed in the native bone. The aim of our study was to compare this null hypothesis being that there was no difference between the two.

## 2 MATERIALS AND METHODS

A retrospective clinical study, based on the files and radiographs of patients, was conducted (Ethics Committee of Erasme Hospital-ULB. Approval P2013 / 296).

### 2.1 Patient Selection

Patients of our clinic who had received implant following a one stage technique, preceded or not by post-extraction alveolar bone preservation using allograft (DFDBA: 300-500 µm), and platelet concentrates were selected (Table **1**). Patients were restricted to those with implant prosthetics in progress or completed, allowing for radiological monitoring.

The following exclusion criteria were applied: the consumption of 20 or more cigarettes/day, taking of bisphosphonates, ongoing chemotherapy treatment, high-risk heart disease and/or an uncontrolled systemic or periodontal disease.

The characteristics considered to be risk factors were collected from patient data to allow for an analysis by subgroups. The study included a total of 84 patients. Among these patients, 247 implants (^®^Nobelbiocare Speedy Groovy in the maxilla and MKIII in the mandible) were placed. Among these implants, 169 were inserted into the native bone (control group), and 78 were placed in the post-extraction alveolar bone filled with allograft and platelet concentrates (test group) (Table **1**). These 247 implants restored 84 mono-radicular teeth and 163 pluri-radicular teeth.

In the test group, the technique used to optimize the maintenance of the post-extraction alveolar bone volume, combining a mixture of particulate bank bone (allograft: DFDBA 300-500 μm) with platelet concentrates, was applied. At the time of the beginning of extractional surgery, platelet concentrates (platelet-rich fibrin) were obtained by centrifugation of blood samples of the patient in 10 ml tubes with no adjuvant anticoagulant, centrifuged at 3000 rpm for 10 minutes, following a protocol described previously [22]. Parts of the centrifuged blood rich in platelets (called buffy-coats) were cut and mixed with 300-500 µm of particulate DFDBA (2 buffy-coats /1.75 cm^3^ DFDBA 300-500 µm). Parts rich in fibrin were pressed manually between gauze to obtain autologous rich-in-fibrin membranes. Atraumatic extractions were realized, and immediately afterward, filling of the socket was performed with the mixture of DFDBA and platelet concentrates. Closure of the sockets was performed using autologous fibrin membrane to cover the filled socket and polyglactin absorbable sutures to close (Vicryl^®^ 3/0).

The implants were placed after a period of 3 to 6 months of healing, according to a surgical procedure in one stage. Implants were placed in each case with the neck precisely positioned at the level of the bone.

### 2.2 Measurement Methods of Peri-Implant Bone Loss

**Fig. (1):**
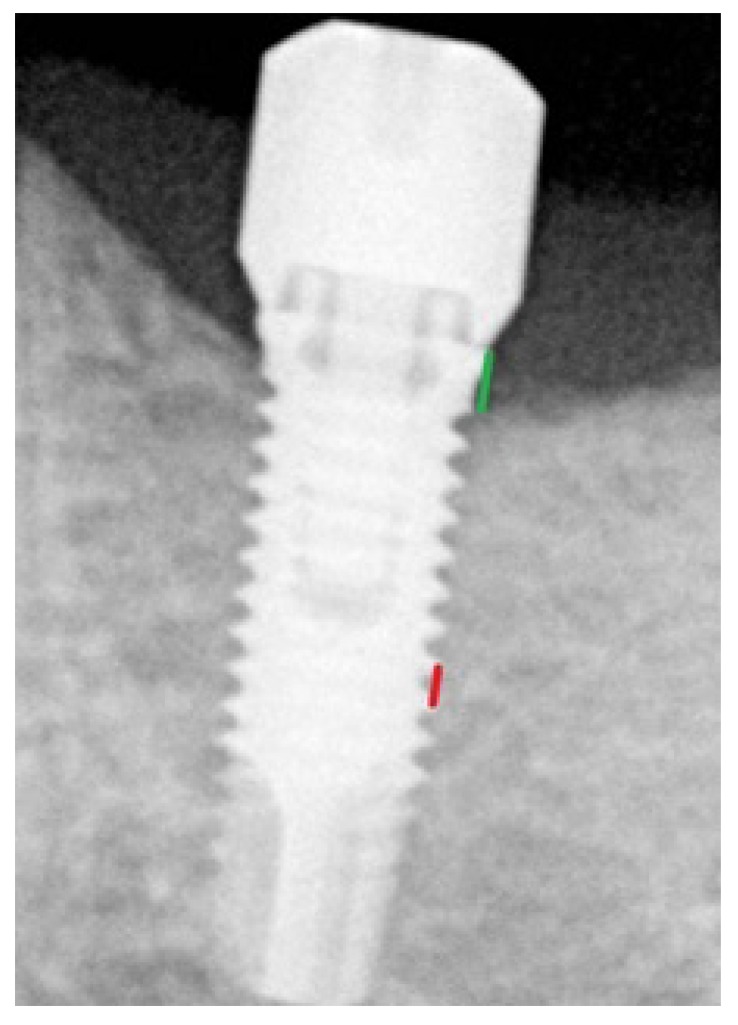
Method of bone loss measurements and of calibration on radiographs. Bone loss (1) was defined as the distance between the top of the neck of the implant (arrow) and bone level in contact with implant (*). Each measurement was calibrated by a rule of three, knowing distance between two threads of the implants (2).

Peri-implant bone loss was measured in the mesial and distal aspects of each implant on panoramic radiographs or on retro-alveolar radiographs with an orthogonal incidence of the X-ray (Fig. **1**), at a period of 6 months after placement of the implant and also after a period of 12 months for all patients who presented for follow-up appointments. These measurements were performed using the “Romexis” X-ray software with accuracy ±0.1 mm. Measurements were performed by two independent operators.

Each radiograph was calibrated. The two types of implants (^®^Nobelbiocare Speedy Groovy in the maxilla and MKIII in the mandible), and their diameters and lengths were recorded. Depending on these parameters, the distance between two threads of the implant possessed a determined value that then allowed for scaling of the radiograph by a rule of three. The peri-implant bone loss could thus be calculated precisely. This loss was defined as the distance between the neck of the implant and the bone level in contact with the implant (Fig. **1**). No radiograph was performed just after the placement of the implant because implants had been placed in each case with neck precisely positioned at the level of the bone. Given the retrospective nature of the study, we were unable to record the bleeding on probing nor the pocket probing deep.

### 2.3 Statistical Analysis

*SPSS* statistical software, version 22, was used. The peri-implant bone losses are presented as the means and standard deviations.

The size of our sample allowed us to use parametric tests. Student’s t-test was performed to compare the peri-implant bone loss in the test group and in the control group at 6 months and 12 months. Student’s t-test was used to determine whether there was a statistically significant difference between the peri-implant bone loss in the maxilla compared to the mandible in the patients with a history of periodontitis now stabilized compared with patients without a periodontal history at periods of 6 months and 12 months among the separate groups: native bone, allograft and global groups.

ANOVA was performed to compare the peri-implant bone loss among the unit, partial, total edentation sites at 6 months and 12 months in the three groups mentioned above.

A pairwise comparison was then performed using the Games-Howell or Bonferroni tests depending on whether we rejected or not the equality of variances.

Finally, we compared the average measurements of the two independent operators by the calculation of correlation coefficients.

A *p*-value <0.05 was considered to indicate a significant statistically level.

## 3 RESULTS

### 3.1 Inter-operator Concordance

Regarding the concordance between the two independent operators, the correlation coefficients near 1 indicated a good degree of association between the measurements of both manipulators (Table **2**).

### 3.2 Peri-Implant Bone Loss

Of the 212 implants evaluated at 6 months, the overall peri-implant bone loss was 0.9 ± 0.7 mm at the mesial level and 0.9 ± 0.8 mm at the distal. At 12 months, the overall peri-implant bone loss on 73 implants was 1 ± 0.65 mm at the mesial level and 1.1 ± 0.7 mm at the distal level (Table **3**).

Of the 140 implants inserted in the native bone at a period of 6 months, the peri-implant bone loss was 1 ± 0.7 mm at the mesial level and 1 ± 0.8 mm at the distal level.

Of the 72 implants placed in an allograft at a period of 6 months, the peri-implant bone loss was 0.8 ± 0.8 mm at the mesial level and 0.8 ± 0.7 mm at the distal level.

Of the 50 implants inserted in the native bone at a period of 12 months, the peri-implant bone loss was 0.95 ± 0.5 mm at the mesial level and 1.1 ± 0.7 mm at the distal level.

Of the 23 implants placed in an allograft at a period of 12 months, the peri-implant bone loss was 1.2 ± 0.9 mm at the mesial level and 1.25 ± 0.8 mm at the distal level (Table **4**).

**Fig. (2):**
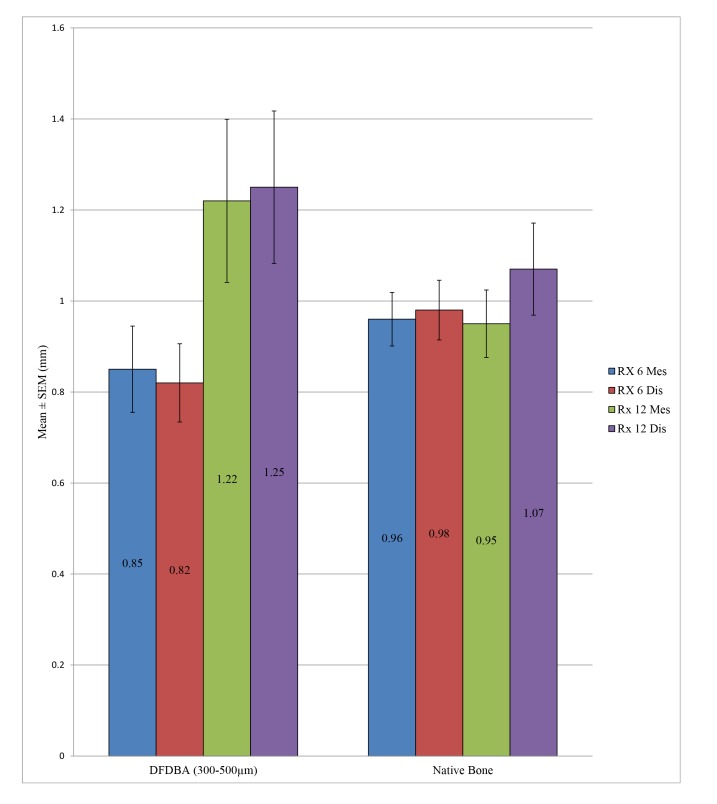
Comparison of the peri-implant bone loss in the test group (DFDBA: 300-500 µm) *versus* the control group (native bone).

Student’s t-test allowed us to note that there was no statistically significant difference between the peri-implant bone loss for the implants placed in the native bone and in an allograft at a time point of 6 months, either at the mesial level (*p* = 0.303) or the distal level (*p* = 0.167). This same conclusion was observed at 12 months both at the mesial level (*p* = 0.183) and the distal level (*p* = 0.337) (Table **5** and Fig. **2**).

### 3.3 Comparison of the Peri-Implant Bone Loss in the Maxilla *Versus* Mandible

**Fig. (3):**
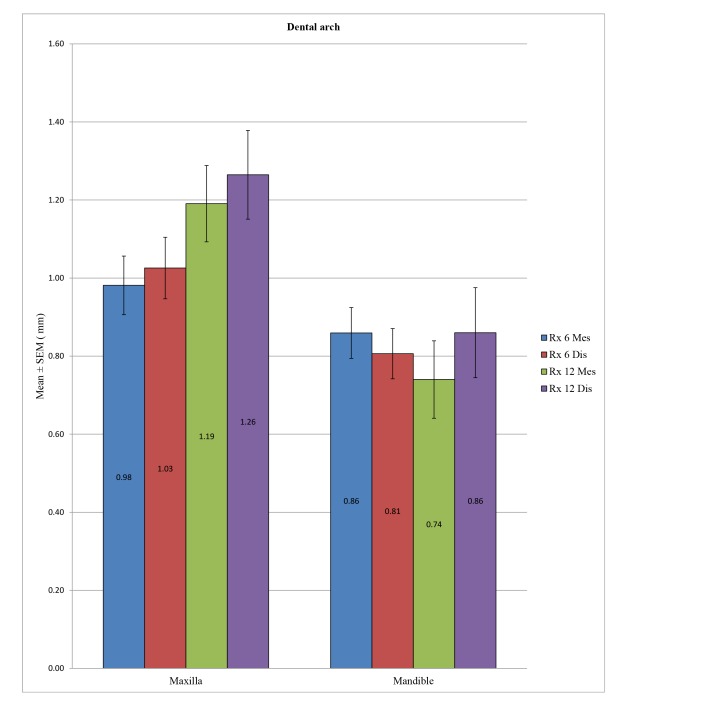
Comparison in the global group (test and control groups taken together) of the peri-implant bone loss in the maxilla *versus* mandible.

We observed a statistically significant difference between the overall peri-implant bone loss in the mandible and in the maxilla at a time point of 6 months at the distal level (*p* = 0.032) and also at 12 months at both the mesial (*p* = 0.004) and distal levels (*p* = 0.026). However, it was not possible to identify a difference at the mesial level at 6 months (*p* = 0.229) (Table **6** and Fig. **3**).

**Fig. (4):**
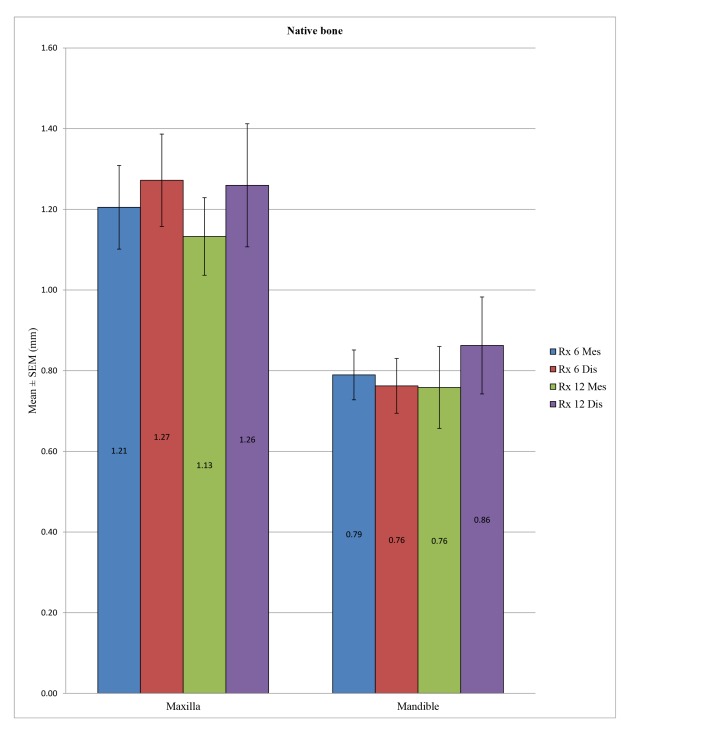
Comparison in the control group of the peri-implant bone loss in the maxilla *versus* mandible.

**Fig. (5):**
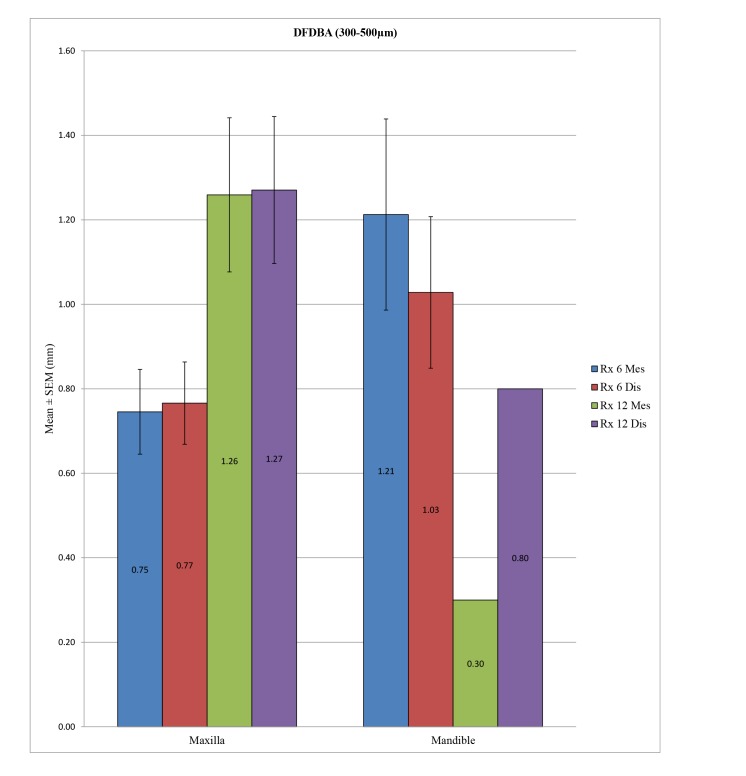
CompaIn the control group, no statistically bone loss in the maxilla *versus* mandible.

In the control group, the peri-implant bone loss was significantly different at the statistical level for the implants placed in the mandible *versus* in the maxilla, both at a 6 months at both the mesial (*p* <0.001) and distal levels (*p* <0.001) and at 12 months at both the mesial (*p* = 0.010) and distal levels (*p* = 0.049). In the test group, a statistically significant difference could not be determined between the mandible and the maxilla at 12 months at the mesial (*p* = 0.285) or distal level (*p* = 0.579) or at 6 months at the distal level (*p* = 0.207), unlike at the mesial level, with *p* = 0.040. (Table **7**; Figs. **4** and **5**). Overall, the maxillary bone loss was greater than the mandibular bone loss, from an average of 0.2 mm at 6 months to 0.4 mm at 12 months.

### 3.4 Comparison of the Peri-Implant Bone Loss in a Patient With and Without Histories of Periodontitis

**Fig. (6):**
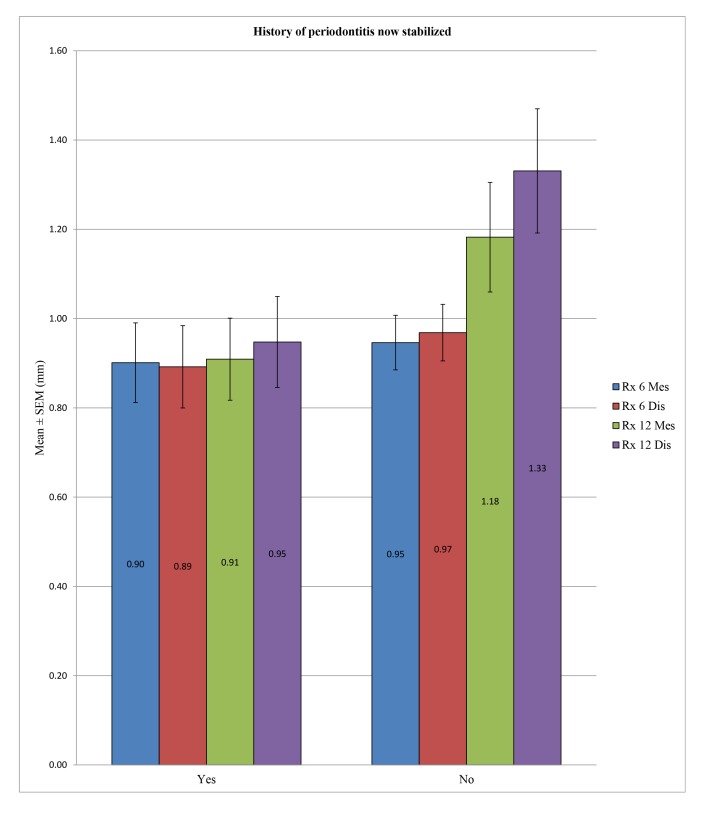
Comparison in the global group (test and control groups taken together) of the peri-implant bone loss in the patients with a history of periodontitis *versus* those without.

No statistically significant difference could be demonstrated for the overall peri-implant bone loss between the patients with a history of periodontitis stabilized and patients without periodontal histories at 6 months at the mesial (*p* = 0.667) and distal levels (*p* = 0.480) or at 12 months at the mesial level (*p* = 0.075), except at 12 months at the distal level, with *p* = 0.027 (Table **8** and Fig. **6**).

**Fig. (7):**
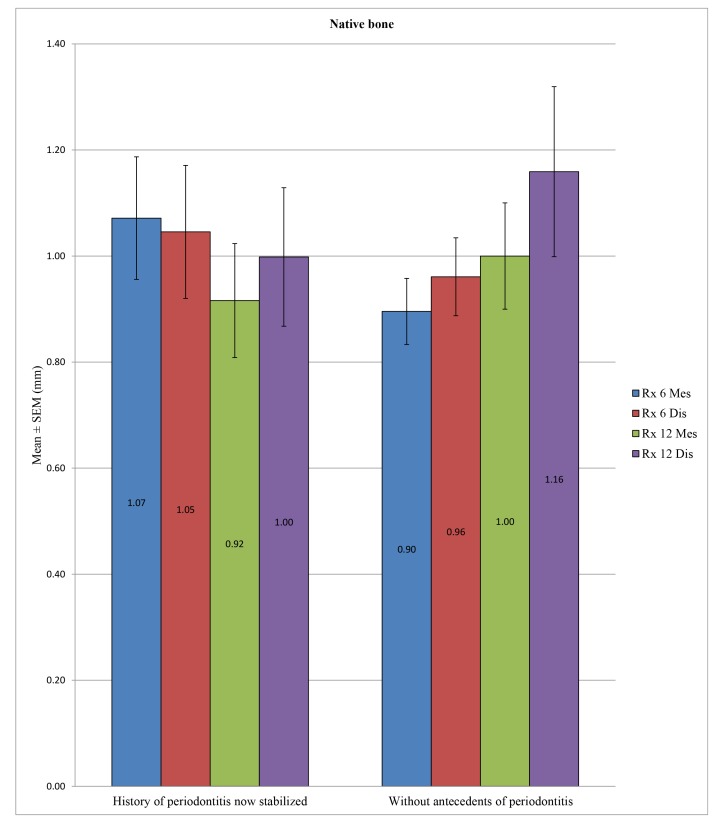
Comparison in the control group of the peri-implant bone loss in the patients with a history of periodontitis *versus* those without.

**Fig. (8):**
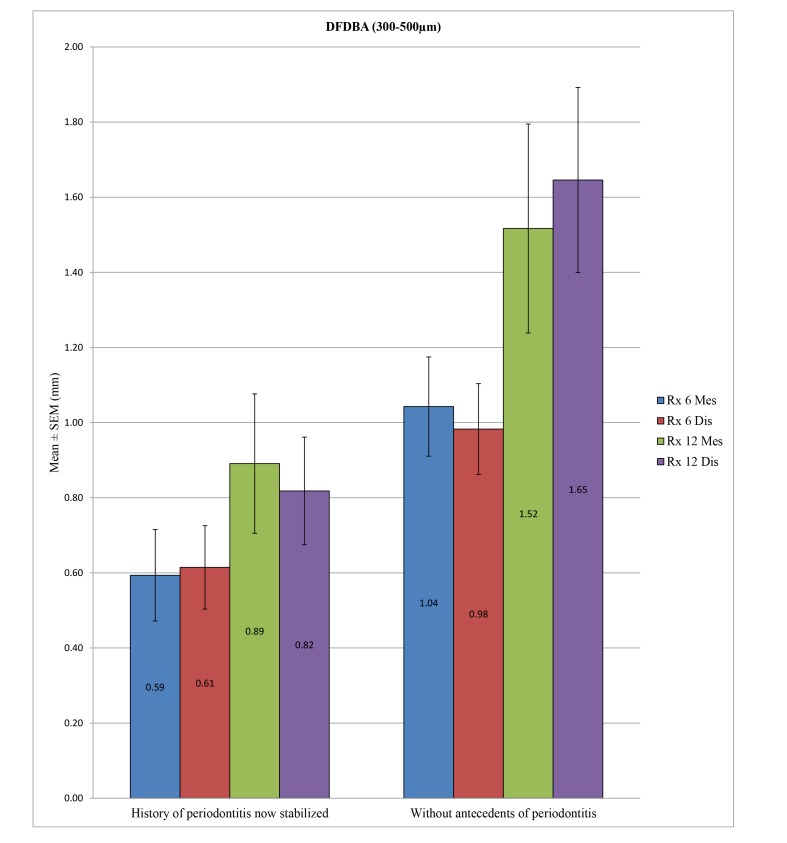
Comparison in the test group of the peri-implant bone loss in the patients with a history of periodontitis *versus* those without.

In the control group, no statistically significant difference was found between the bone loss in patients with a history of periodontitis now stabilized compared to patients without periodontal histories after 6 months at the mesial (*p* = 0.184) and distal levels (*p* = 0.562) or after 12 months at the mesial (*p* = 0.579) and distal levels (*p* = 0.436). In the test group, the difference in peri-implant bone loss was statistically significant between these two groups of patients mentioned above at 6 months at the mesial (*p* = 0.018) and distal levels (*p* = 0.033) or at 12 months at the distal level (*p* = 0.010), unlike at the mesial level, with *p* = 0.081. (Table **9**; Figs. **7** and **8**)

Regardless of the test group, we observed no significant difference between the two groups of patients.

### 3.5 Comparison of the Peri-Implant Bone Loss in Unitary Edentation *Versus* Partial Edentation *Versus* Total Edentation

**Fig. (9):**
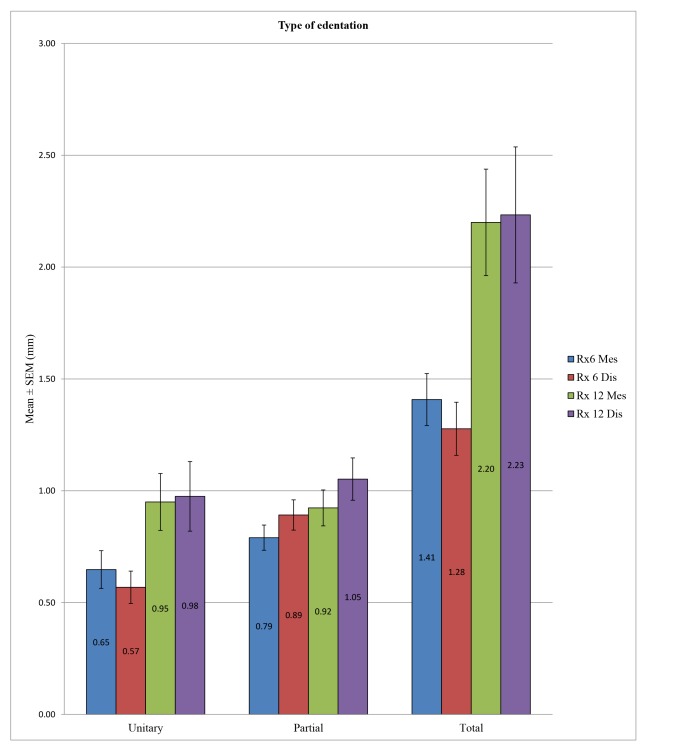
Comparison in the global group (test and control groups taken together) of the peri-implant bone loss in unitary edentation *versus* partial edentation *versus* total edentation.

ANOVA allowed us to find a statistically significant difference in overall peri-implant bone loss among the various types of edentation at 6 months at the mesial (*p* <0.001) and distal levels (*p* <0.001) and also at 12 months at the mesial (*p* <0.001) and distal levels (*p* <0.001). More precisely, at 6 months, we noted at the mesial level differences between the unitary and total edentation (*p* <0.001) and between the partial and total edentation (*p* <0.001) but no difference between the unitary and partial edentation (*p* = 0.342). At 6 months, at the distal level, there was a difference between the unitary and partial edentation (*p* = 0.004), between the unitary and total edentation (*p* <0.001), and between the partial and total edentation *p* = 0.016. Moreover, at 12 months, we noted at the mesial and distal levels the absence of differences between the unitary and partial edentation (*p* = 1) and a difference between the unitary and total edentation (*p* <0.001) and between the partial and total edentation (*p* <0.001) (Table **10** and Fig. **9**).

**Fig. (10):**
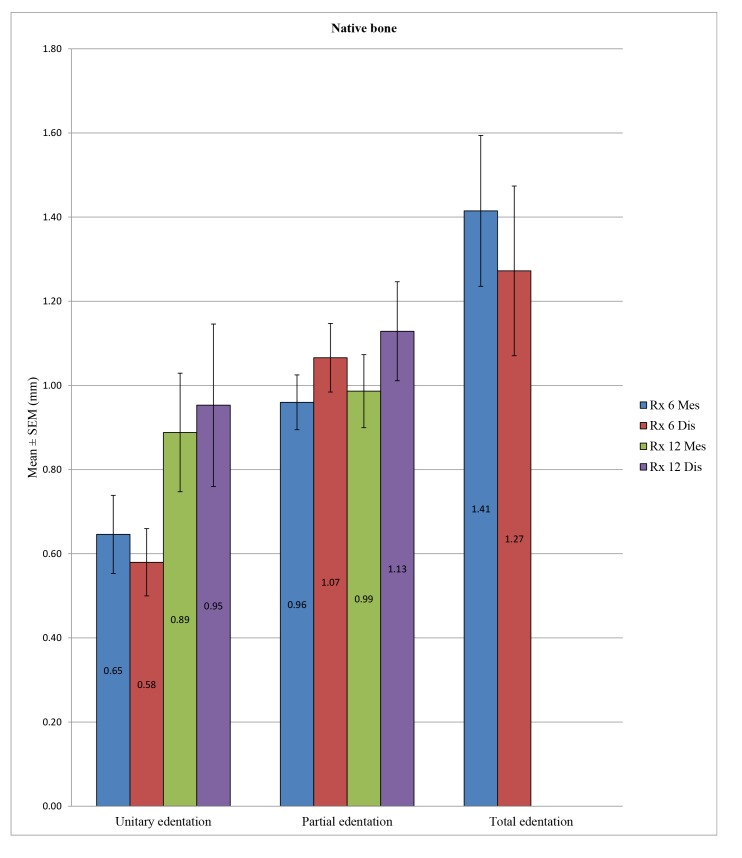
Comparison in the control group of the peri-implant bone loss in unitary edentation *versus* partial edentation *versus* total edentation.

**Fig. (11):**
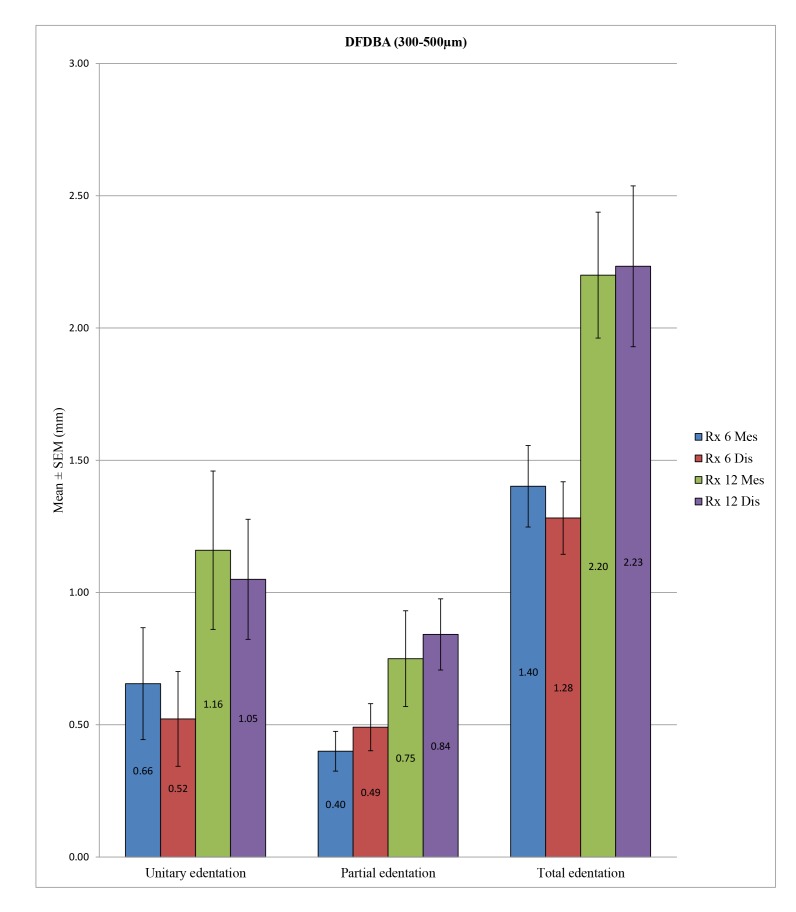
Comparison in the test group of the peri-implant bone loss in unitary edentation *versus* partial edentation *versus* total edentation.

In the control group, there was a statistically significant difference in peri-implant bone loss among the three types of edentation at 6 months (*p* = 0.001) at the mesial and distal levels (*p* <0.001), but such a difference was not observed at 12 months at the mesial (*p* = 0.536) and distal levels (*p* = 0.416). More specifically, at 6 months, we observed a difference between the unitary and partial edentation at the mesial level (*p* = 0.019), as well as at the distal level (*p* <0.001), and between the unitary and total edentation at the mesial (*p* = 0.001) and distal levels (*p* = 0.008), but between the partial and total edentation, differences could not be found at the mesial (*p* = 0.058) or distal level (*p* = 0.613). In the test group, we note a statistically significant difference in peri-implant bone loss among the different types of edentation at 6 months at the mesial (*p* <0.001) and distal levels (*p* <0.001) and also at 12 months at the mesial (*p* = 0.001) and distal levels (*p* <0.001). More precisely, at 6 months, we found differences between the unitary and total edentation at the mesial (*p* = 0.028) and distal levels (*p* = 0.006) and between the partial and total edentation at the mesial (*p* <0.001) and distal levels (*p* <0.001), but we did not observe differences between the partial and unitary edentation at mesial (*p* = 0.514) or distal level (*p* = 1). At 12 months, we found differences between the unitary and total edentation at the mesial (*p* = 0.037) and distal levels (*p* = 0.007) and between the partial and total edentation at the mesial (*p* <0.001) and distal levels (*p* <0.001), but we did not note differences between the partial and unitary edentation at the mesial (*p* = 0.697) or distal levels (*p* = 1) (Table **11**; Figs. **10** and **11**).

Despite some exceptions, the general trend was that there is a significant difference in bone loss between the unitary and total edentation (difference on average of 0.7 mm at 6 months and 1.2 mm at 12 months) and between the partial and total edentation (difference on average of 0.5 mm at 6 months and 1.2 mm at 12 months) but no differences between the partial and unitary areas (Table **12**).

## 4 DISCUSSION

It is difficult to compare the results of this study with those of other studies because there have been few studies of implant behavior in bone grafts and because there is a diversity of biomaterials and techniques used for alveolar preservation. Our study was focused on the peri-implant bone loss as a criterion of success. Then, the discussion is concentrated on the comparison of peri-implant bone loss in our study and in the other studies with different types of post-extraction alveolar bone conservation techniques.

According to the study by Barone *et al.* in 2012 [38], the average peri-implant bone loss at 3 years of follow-up was 1.02 ± 0.3 mm for the group without alveolar conservation and 1.00 ± 0.2 mm for the group with tooth sockets preserved using xenograft (pig bone). Furthermore, the authors did not observe significant differences in the marginal bone loss between the two groups at 1 year, 2 years or 3 years.

Crespi *et al*. compared in 2009 [39] the peri-implant bone loss with three biomaterials: Magnesium enriched in Hydroxyapatite (MHA), Calcium Sulfate (CS) and a xenograft (Pig Bone = PB). It emerged that there was no statistically significant difference at level of the bone loss at either the mesial or distal level among the groups after a period of 24 months. The average peri-implant bone loss after 24 months was 0.21 ± 0.09 mm for the MHA group, 0.13 ± 0.09 mm for the CS group, and 0.16 ± 0.08 mm for the PB group.

Patel *et al*. performed in 2012 [40] implant placement in tooth sockets preserved with synthetic bone graft (Straumann Bone Ceramic = SBC) or xenograft (from Bovine Bone = DBBM) and a barrier of collagen. A radiological assessment was also performed. At one year after loading, the authors did not observe any statistically significant differences in a peri-implant bone loss at the mesial and distal levels. The average bone loss after 1 year was 3.58 ± 1.02 mm at the mesial level and 3.28 ± 1.03 mm at the distal level for the SBC group and 3.71 ± 0.77 mm at the mesial level and 3.58 ± 0.78 mm at the distal level for the DDBM group.

Block *et al*. in 2002 [41] placed 22 implants, of which 3 were inserted immediately after the extraction of single-rooted tooth with a human mineralized cancellous bone complement and the remainder of which were placed in a tooth socket preserved with an allograft (human mineralized cancellous bone). The radiological measurements at 4 months after the implant placement revealed an average bone loss of 0.51 ± 0.41 mm at the mesial level and 0.48 ± 0.53 mm at the distal level.

Koutouzis *et al*. in a retrospective and radiological study [42], found that the average peri-implant bone loss at a period of 12 months of follow-up was 0.15 ± 0.33 mm in the group that had alveolar bone conservation with an allograft (DFDBA) and 0.16 ± 0.32 mm in the group without alveolar bone preservation with no significant difference.

These articles showed that there was no difference in the peri-implant bone losses between tooth sockets preserved with different biomaterials and with native bone.

In our study including 247 implants in which a part was inserted into native bone and the remainder within a tooth socket preserved with DFDBA 300-500 µm and platelet concentrates, no statistically significant differences were found after radiological evaluation of the peri-implant bone loss at 6 months and 12 months at both the mesial and distal levels. These results were similar to those reported in some previous studies [38-42] but different from those cited in the study of Theofilos Koutouzis *et al*. [42], in which DFDBA was used as an alveolar filling material. However, in the latter study, tooth sockets were covered with collagen membranes, and the implants had a design of type “platform switching” that was supposed to reduce marginal bone loss [42]. Regarding differences in the protocol of alveolar preservation and type of implant, it is difficult to compare them rigorously with our study. We observed a significant difference between maxillary and mandibular bone loss for the control group; however, such a difference could not be noted in the test group except at the mesial level at 6 months. In the study by Theofilos Koutouzis *et al*. [42], no significant differences were found for maxillary or mandibular bone loss, unlike our results, in which the overall bone loss was significantly greater in the maxilla than in the mandible at 12 months on both sides and at 6 months only at the distal level.

Our results allowed us to note a lack of significant differences between peri-implant bone loss in the patients with a history of periodontitis now stabilized compared to patients without periodontal histories at 6 months and 12 months in the overall and control groups, while a difference existed in the test group (except at 12 months at the mesial level). However, in this test group, contrary to what one might think, the bone loss was greater in the patients without a history of periodontal disease. This finding can be explained by most of these implants being placed in patients who were completely toothless and therefore who experienced greater bone loss. In the study of Rinke *et al*. in 2011 [43], a significant association could not be found between the history of periodontitis and an increased prevalence of peri-implantitis. In contrast, Karoussis *et al*. in 2003 [44] and Hardt *et al*. in 2002 [45] observed a significantly increased bone loss in patients with histories of periodontitis.

Concerning the sites of edentation, a global trend emerged because the peri-implant bone loss was significantly different between the unitary and total edentation (6 months: 0.6 mm *versus* 1.3 mm/12 months: 1 mm *versus* 2.2 mm) and between the partial and total edentation (6 months: 0.8 mm *versus* 1.3 mm/12 months: 1 mm *versus* 2.2 mm); however, there was no difference at the level of bone loss between the unitary and partial edentation (6 months: 0.6 mm *versus* 0.8 mm/12 months: 0.96 mm *versus* 0.98 mm) except in the test group at 6 months. These findings were in agreement with those of previous studies, including Berglundh *et al*. in 2002 [7]. Fransson *et al*. in 2005 [46] noted that the bone loss was greater in total edentation compared to unitary edentation, in which the bone loss was minimal. They also hypothesized that implants placed in the partial and total edentation yielded the same results.

Oral biofilm with the accumulation of the microorganisms is the major factor responsible for the peri-implant bone loss. A predominance of anaerobic bacteria is present when there is a peri-implantitis. The microbiota associated with peri-implantitis is different from the one from the periodontitis. Indeed, the surroundings of the implant in titanium provide a different environment than a tooth and therefore the bacteria around the implants are different. The flora is similar between peri-implantitis and chronic periodontitis, but bacteria like *Staphylococcus aureus, Enerobacteriaceae, Candida albicans* are a frequent finding in peri-implantitis. If the amount of bacteria is too important, there will be an infection and so the biofilm must be removed with various techniques and adjuvants. Following several studies, the fully edentulous patients have a microbiota that has less pathogenic plaque compared with partially edentulous subjects [12, 14, 15].

## CONCLUSION

Considering the results of this study, we can conclude that the implants placed in tooth sockets preserved with a mix of particulated allogenic bone (DFDBA: 300-500 μm) and platelet concentrates behave similarly to implants inserted in native bone regarding the peri-implant bone loss, and that peri-implant bone loss remains inferior to tolerated bone loss to be able to consider implant success.

## Figures and Tables

**Table 1:** Table of effective.

–	N Implants	N Patients
Total	247	84
Native bone (control group)	169	
DFDBA (300-500 µm) + platelet concentrates (test group)	78	

**Table 2:** Comparison of the peri-implant bone loss between the two independent operators with correlation coefficients.

–		N	Mean (mm)	SD (mm)	Correlation Coefficients
Pair 1	Rx 6 Mes J	212	0.9255	0.7352	0.898
	Rx 6 Mes A	212	0.8901	0.7287	
Pair 2	Rx 6 Dis J	212	0.9252	0.7623	0.941
	Rx 6 Dis A	212	0.8807	0.7458	
Pair 3	Rx 12 Mes J	212	1.0363	0.6542	0.812
	Rx 12 Mes A	73	0.9808	0.6434	
Pair 4	Rx 12 Dis J	73	1.1260	0.7434	0.947
	Rx 12 Dis A	73	1.0178	0.7036	

**Table 3:** Peri-implant bone loss in the global group (test and control groups taken together).

–	Rx 6 Més	Rx 6 Dis	Rx 12 Més	Rx 12 Dis
N	212	212	73	73
Mean (mm)	0.925	0.925	1.036	1.126
SD (mm)	0.735	0.762	0.654	0.743

**Table 4:** Peri-implant bone loss in the native bone and in the DFDBA.

–		Rx 6 Més	Rx 6 Dis	Rx 12 Més	Rx 12 Dis
Native bone	N	140	140	50	50
	Mean (mm)	0.9646	0.9771	0.9530	1.0690
	SD (mm)	0.6966	0.7760	0.5242	0.7155
DFDBA (300-500 µm)	N	72	72	23	23
	Mean (mm)	0.8493	0.8243	1.2174	1.2500
	SD (mm)	0.8046	0.7296	0.8595	0.8033

**Table 5:** Comparison of peri-implant bone loss in DFDBA(300-500 µm) *versus* in native bone.

–		N	Mean (mm)	SD (mm)	*P*-value
Rx 6 Mes	DFDBA (300-500 µm)	72	0.8493	0.8046	0.303
	Native bone	140	0.9646	0.6966	
Rx 6 Dis	DFDBA (300-500 µm)	72	0.8243	0.7296	0.167
	Native bone	140	0.9771	0.7760	
Rx 12 Mes	DFDBA (300-500 µm)	23	1.2174	0.8595	0.183
	Native bone	50	0.9530	0.5242	
Rx 12 Dis	DFDBA (300-500 µm)	23	1.2500	0.8033	0.337
	Native bone	50	1.0690	0.7155	

**Table 6:** Comparison in the global group (test and control groups taken together) of the peri-implant bone loss in the maxilla *versus* mandible.

–	Dental Arch	N	Mean (mm)	SD (mm)	*P*-value
Rx 6 Mes	Maxilla	115	0.9813	0.8049	0.229
	Mandible	97	0.8593	0.6407	
Rx 6 Dis	Maxilla	115	1.0257	0.8454	0.032*
	Mandible	97	0.8062	0.6339	
Rx 12 Mes	Maxilla	48	1.1906	0.6776	0.004*
	Mandible	25	0.7400	0.4958	
Rx 12 Dis	Maxilla	48	1.2646	0.7872	0.026*
	Mandible	25	0.8600	0.5766	

**Table 7:** Comparison in the control and test groups of the peri-implant bone loss in the maxilla *versus* mandible.

Native Bone	Dental Arch	N	Mean (mm)	SD (mm)	*P*-value
Rx 6 Mes	Maxilla	59	1.2051	0.7968	<0.001*
	Mandible	81	0.7895	0.5560	
Rx 6 Dis	Maxilla	59	1.2720	0.8799	<0.001*
	Mandible	81	0.7623	0.6115	
Rx 12 Mes	Maxilla	26	1.1327	0.4905	0.010*
	Mandible	24	0.7583	0.4977	
Rx 12 Dis	Maxilla	26	1.2596	0.7782	0.049*
	Mandible	24	0.8625	0.5889	
**DFDBA (300-500 µm)**	**Dental Arch**	**N**	**Mean (mm)**	**SD (mm)**	***P*-value**
Rx 6 Mes	Maxilla	56	0.7455	0.7504	0.040*
	Mandible	16	1.2125	0.9049	
Rx 6 Dis	Maxilla	56	0.7661	0.7289	0.207
	Mandible	16	1.0281	0.7174	
Rx 12 Mes	Maxilla	22	1.2591	0.8556	0.285
	Mandible	1	0.3000	–	
Rx 12 Dis	Maxilla	22	1.2705	0.8160	0.579
	Mandible	1	0.8000	–	

**Table 8:** Comparison in the global group (test and control groups taken together) of the peri-implant bone loss in patients with a history of periodontitis now stabilized *versus* those without.

–		History of Periodontitis now Stabilized	N	Mean (mm)	SD (mm)	*P*-value
Rx 6 Mes	Yes		87	0.9011	0.8319	0.667
	No		119	0.9462	0.6673	
Rx 6 Dis	Yes		87	0.8920	0.8601	0.480
	No		119	0.9685	0.6910	
Rx 12 Mes	Yes		39	0.9090	0.5742	0.075
	No		34	1.1824	0.7162	
Rx 12 Dis	Yes		39	0.9474	0.6367	0.027*
	No		34	1.3309	0.8113	

**Table 9:** Comparison in the control and test groups of the peri-implant bone loss in patients with a history of periodontitis now stabilized *versus* those without.

**Native Bone**		**History of Periodontitis now Stabilized**		**N**	**Mean (mm)**	**SD (mm)**		***P*-value**	
Rx 6 Mes		Yes		56	1.0714	0.8653		0.184	
		No		78	0.8955	0.5506			
Rx 6 Dis		Yes		56	1.0455	0.9386		0.562	
		No		78	0.9609	0.6486			
Rx 12 Mes		Yes		28	0.9161	0.5688		0.579	
		No		22	1.0000	0.4701			
Rx 12 Dis		Yes		28	0.9982	0.6910		0.436	
		No		22	1.1591	0.7519			
**DFDBA (300-500 µm)**		**History of periodontitis now stabilized**		**N**	**Mean (mm)**	**SD (mm)**		***P*-value**	
Rx 6 Mes		Yes		31	0.5935	0.6777		0.018*	
		No		41	1.0427	0.8459			
Rx 6 Dis		Yes		31	0.6145	0.6180		0.033*	
		No		41	0.9829	0.7737			
Rx 12 Mes		Yes		11	0.8909	0.6156		0.081	
		No		12	1.5167	0.9637			
Rx 12 Dis		Yes		11	0.8182	0.4750		0.010*	
		No		12	1,6458	0,8532			

**Table 10:** Comparison in the global group (test and control groups taken together) of the peri-implant bone loss in unitary edentation *versus* partial edentation *versus* total edentation.

–	Type of Edentation	N	Mean (mm)	SD (mm)	*P*-value ANOVA	Pairs *P*-value: Games Howell/Bonferroni
Rx 6 Mes	Unitary	46	0.6478	0.5718	<0.001	U-P: 0.342
	Partial	109	0.7904	0.5880		U-T: <0.001*
	Total	57	1.4079	0.8782		P-T: <0.001*
Rx 6 Dis	Unitary	46	0.5685	0.4908	<0.001	U-P: 0.004*
	Partial	109	0.8917	0.7059		U-T: <0.001*
	Total	57	1.2772	0.8954		P-T: 0.016*
Rx 12 Mes	Unitary	22	0.9500	0.5966	<0.001	U-P: 1.000
	Partial	45	0.9233	0.5389		U-T: <0.001*
	Total	6	2.2000	0.5831		P-T: <0.001*
Rx 12 Dis	Unitary	22	0.9750	0.7299	<0.001	U-P: 1.000
	Partial	45	1.0522	0.6348		U-T: <0.001*
	Total	6	2.2333	0.7448		P-T: <0.001*

**Table 11:** Comparison in the control and test groups of the peri-implant bone loss in unitary edentation *versus* partial edentation *versus* total edentation.

Native Bone	Type of Edentation	N	Mean (mm)	SD (mm)	*P*-value ANOVA	Pairs *P*-value: Games Howell/Bonferroni
Rx 6 Mes	Unitary	37	0.6459	0.5650	<0.001	U-P: 0.019*
	Partial	76	0.9599	0.5676		U-T: 0.001*
	Total	27	1.4148	0.9315		P-T: 0.058
Rx 6 Dis	Unitary	37	0.5797	0.4861	<0.001	U-P: <0.001*
	Partial	76	1.0658	0.7104		U-T: 0.008*
	Total	27	1.2722	1.0478		P-T: 0.613
Rx 12 Mes	Unitary	17	0.8882	0.5808	0.536	–
	Partial	33	0.9864	0.4986		
	Total	0				
Rx 12 Dis	Unitary	17	0.9529	0.7954	0.416	–
	Partial	33	1.1288	0.6758		
	Total	0				
**DFDBA (300-500 µm)**	**Type of edentation**	**N**	**Mean (mm)**	**SD (mm)**	***P*-value ANOVA**	**Pairs *P*-value: Games Howell/Bonferroni**
Rx 6 Mes	Unitary	9	0.6556	0.6346	<0.001	U-P: 0.514
	Partial	33	0.4000	0.4316		U-T: 0.028*
	Total	30	1.4017	0.8434		P-T: <0.001*
Rx 6 Dis	Unitary	9	0.5222	0.5374	<0.001	U-P: 1.000
	Partial	33	0.4909	0.5113		U-T: 0.006*
	Total	30	1.2817	0.7509		P-T: <0.001*
Rx 12 Mes	Unitary	5	1.1600	0.6693	0.001	U-P: 0.697
	Partial	12	0.7500	0.6274		U-T: 0.037*
	Total	6	2.2000	0.5831		P-T: <0.001*
Rx 12 Dis	Unitary	5	1.0500	0.5074	<0.001	U-P: 1.000
	Partial	12	0.8417	0.4660		U-T: 0.007*
	Total	6	2.2333	0.7448		P-T: <0.001*

**Table 12:** Summary of the results.

**Peri-Implant Bone Loss**	
**DFDBA (300-500 µm)**	**Native bone**
6 months: 0.83 ± 0.77 mm/12 months: 1.23 ± 0.83 mm	6 months: 0.97 ± 0.74 mm/12 months: 1.01 ± 0.62 mm
No significant difference (*p* > 0.05)	
**With/without history of periodontitis**	
No significant difference (*p* > 0.05)	
**Maxilla/Mandible**	
Significant difference (*p* < 0.05): maxilla > mandible	
**Type of edentation**	
Significant difference (*p* < 0.05): total > partial = unitary	
